# Mammalian Host-Versus-Phage immune response determines phage fate *in vivo*

**DOI:** 10.1038/srep14802

**Published:** 2015-10-06

**Authors:** Katarzyna Hodyra-Stefaniak, Paulina Miernikiewicz, Jarosław Drapała, Marek Drab, Ewa Jończyk-Matysiak, Dorota Lecion, Zuzanna Kaźmierczak, Weronika Beta, Joanna Majewska, Marek Harhala, Barbara Bubak, Anna Kłopot, Andrzej Górski, Krystyna Dąbrowska

**Affiliations:** 1 Bacteriophage Laboratory, Institute of Immunology and Experimental Therapy, Polish Academy of Sciences, Weigla 12, 53-114 Wrocław, Poland; 2 Institute of Computer Science, Wrocław University of Technology, Wyb. Wyspiańskiego 27, 50-370, Wrocław, Poland; 3 USI, Unit of Nanostructural Bio-Interactions, Department of Immunology of Infectious Diseases, Institute of Immunology and Experimental Therapy, Polish Academy of Sciences, Weigla 12, 53-114 Wroclaw, Poland

## Abstract

Emerging bacterial antibiotic resistance draws attention to bacteriophages as a therapeutic alternative to treat bacterial infection. Examples of phage that combat bacteria abound. However, despite careful testing of antibacterial activity *in vitro*, failures nevertheless commonly occur. We investigated immunological response of phage antibacterial potency *in vivo*. Anti-phage activity of phagocytes, antibodies, and serum complement were identified by direct testing and by high-resolution fluorescent microscopy. We accommodated the experimental data into a mathematical model. We propose a universal schema of innate and adaptive immunity impact on phage pharmacokinetics, based on the results of our numerical simulations. We found that the mammalian-host response to infecting bacteria causes the concomitant removal of phage from the system. We propose the notion that this effect as an indirect pathway of phage inhibition by bacteria with significant relevance for the clinical outcome of phage therapy.

Emerging antibiotic resistance in bacteria has rekindled the old idea that bacterial viruses or phages could have clinical utility^1^. Phages can kill bacteria^2^,^3^,^4^,^5^,^6^. However, despite careful phage selection even when using the newest genome analyses and testing phage antibacterial activity *in vitro* a reliable system relying on phage to combat bacterial infection in mammalian hosts has not been feasible^7^. This state-of-affairs raises the possibility that the infected host could be at least in part responsible for bacterial phage resistance. Phage therapy applied *in vivo* is likely to lead to interactions with the host’s immune system; such interactions could be relevant for the clinical outcome^5^,^8^,^9^,^10^.

Innate immunity represents non-specific reaction to foreign objects recognized by universal molecular patterns associated with pathogens, such as pathogen-associated molecular patterns (PAMPs). Bacterial lipopolysaccharide (LPS), an endotoxin found on the bacterial cell membrane of a bacterium, is considered to be the prototypical PAMP. They are recognized by Toll-like receptors (TLRs) and other pattern recognition receptors (PRRs) in both plants and animals. A vast array of different types of molecules can serve as PAMPs, including glycans and glycoconjugates. They activate the cells that release signaling molecules such as cytokines that mediate the signal of activation to other elements of the immune system. Activation of innate immunity is related to inflammation^11^,^12^,^13^. Adaptive immunity response must be ‘trained’ before it can recognize a selected aim, but thereby it is highly specific and asserts an immunological memory^14^,^15^. Importantly, innate immunity and adaptive immunity elements are tightly linked into one, consistent system. The elements cooperate, stimulate, and/or control each other.

Mathematical modeling and numerical simulations are a potent tool for predicting the outcomes of phage therapy. This approach combines elements of the ‘classic’ pharmacokinetics with population and evolutionary dynamics of self-replicating agents, such as phage^16^,^17^,^18^,^19^,^20^. As a result, phage pharmacokinetics and the resulting effectiveness of phage therapy could be theoretically analyzed by means of mathematical models that allow for computer simulations. Because of the complexity of analysis^17^, simulations have not yet been used for modeling reciprocal dependencies between phage, mammalian host immunity, and bacteria. However, the urgency of such complex physiological-process analyses as occur *in vivo* has been noted and listed among important directions for future studies^19^,^20^.

We investigated the responses to *Pseudomonas* phage F8 and T4 in the mouse to understand how adaptive and innate immunity shape phage pharmacokinetics. We used microbiological methods to detect phage activity in tissues. Additionally, we used direct detection of phage inside immune cells (phagocytes). To detect the 100 nm-sized phage particles, we implemented super-resolution microscopy or ultra-microscopy. Fluorescence-labeled genetically encoded proteins expressed in phage allow the viral particles to be traced within the cell. The limitations of resolution allow only a subset of confocal microscopy techniques to be applied assuming small phage dimensions. Under circumstances of individual phage clustering into larger groups that collectively exceed the resolving limit of classical confocal microscope, the benefits of spectral unmixing were exploited to assure the distinction between the phage signal and the auto-fluorescence noise. The correlated light-electron microscopy has particularly benefited from super-resolution techniques that fill the resolution gap to detect phage uptake within the 3-dimensional context of the cell. We utilized three imaging techniques in this study. Confocal microscopy resolution is limited to 150 nm; super-resolution microscopy features resolving power below 50 nm; scanning electron microscopy can record phage details within a resolution of 5 nm or better. Here, we report the patterns of phage uptake by macrophages.

## Results

### Innate immunity response to phag**e**

Innate immunity is boosted during bacterial infection^21^. However, the impact of innate immunity on the viability of phage cannot be studied directly in infection models, since phage propagate in bacteria. We addressed this problem by inducing systemic inflammatory response syndrome (SIR) with lipopolysaccharide (LPS), a well-known pathogen-associated molecular pattern (PAMP). LPS activated an alerted anti-bacterial inflamed state, which mimicked systemic infection, including fever, leukocytosis, and acute inflammatory responses (Supplementary Fig. 1), but without the presence of living bacteria. Although bacteria resistant to the phage could also have been used with no resulting phage propagation, infection models present more confounding variables.

**Figure 1:**
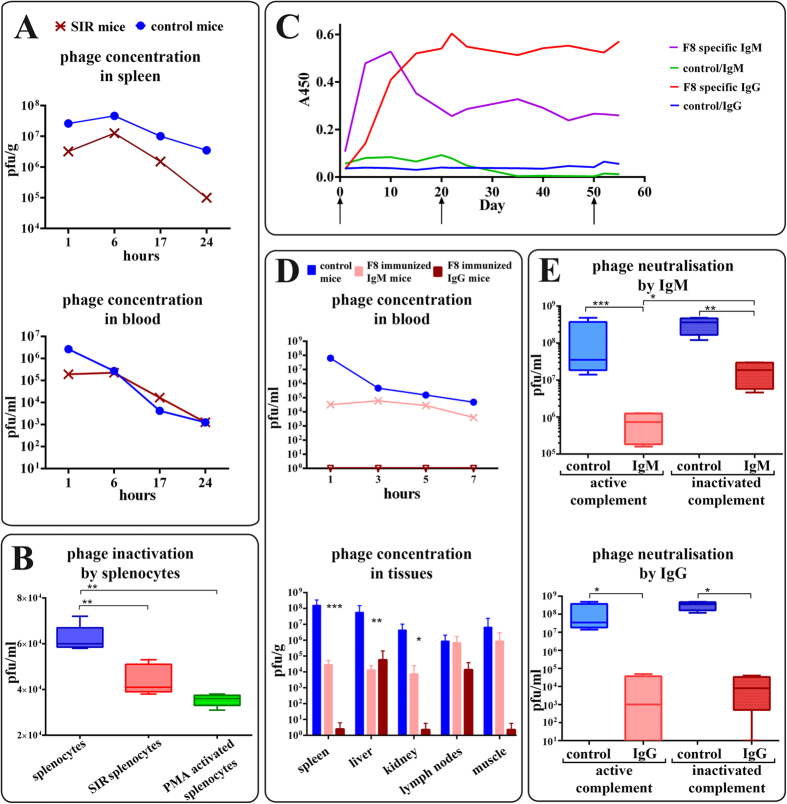
Innate and adaptive immune response to phage impact phage viability. **(A)** Tissue concentration of active F8 phage is lowered in mice with a systemic inflammatory response (SIR). SIR was induced in mice (N = 7) by LPS (see: Supplementary). Bacteriophage F8 was injected i.p 10^9^pfu/mouse. Repeated 5 times, exemplary experiment presented, statistics: ANOVA. **(B)** Activated spleen macrophages neutralize F8 bacteriophage more efficiently. Isolated splenocytes were incubated (N = 5) with F8 phage for 2 h, 37 °C; viable phage was detected in the culture supernatant; ‘splenocytes’- normal splenocytes, ‘SIR splenocytes’- splenocytes from SIR-mice, ‘PMA activated splenocytes’- normal splenocytes induced by phorbolmyristate acetate. Repeated 3 times, exemplary experiment presented, statistics: ANOVA; *p < 0.05, **p < 0.005, ***p < 0.0005. **(C)** F8 phage induces specific IgM and IgG antibodies in a mouse model. Mice (N = 7) were challenged with F8 subcutaneously 1 × 10^10^pfu/mouse (control: PBS) on days 0, 20 and 50 (arrows). F8-specific IgM and IgG were detected in sera by ELISA. Repeated 2 times, exemplary experiment presented, statistics: ANOVA. **(D)** Tissue concentration of active F8 phage is decreased in mice pre-immunized specifically to the phage. F8 phage circulation (3 × 10^9^pfu/mouse i.p.) was compared between pre-immunized and control mice (N = 7). Repeated 3 times, exemplary experiment presented, statistics: ANOVA *p < 0.05, **p < 0.005, ***p < 0.0005. **(E)** Specific IgM and IgG antibodies inactivate F8 phage. F8-specific IgM- and IgG-rich serum isolated from mice (N = 7) were incubated for 1.5 hours at 37 °C; phage activity was detected by plating. Repeated 4 times, exemplary experiment presented, statistics: ANOVA *p < 0.05, **p < 0.005, ***p < 0.0005.

**Figure 2:**
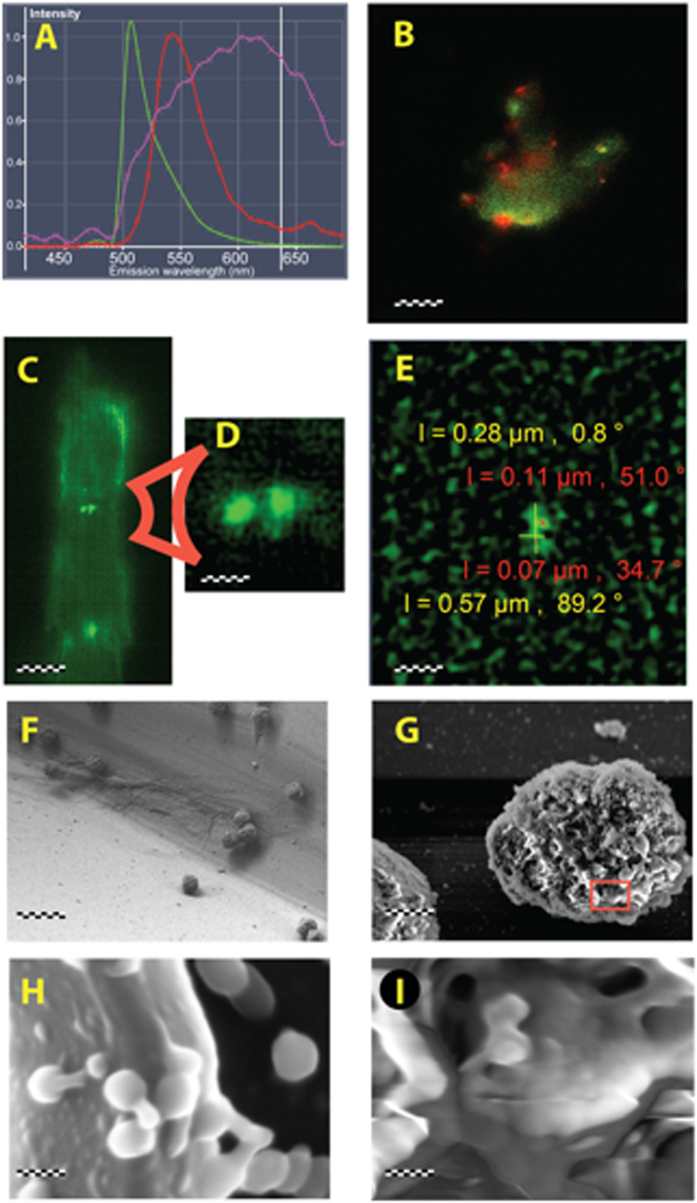
Visualization of bacteriophage phagocytosis in spleen macrophages up-taking the model phage. The particles of GFP-expressing bacteriophage are visible in discrete locations of the macrophage – as clusters of viral particles. Spectral unmixing confocal microscopy of GFP variants (**A,B**) powered by super-resolution microscopy (structural illumination mode, (**B**–**D**)) of macrophages exposed to phages. A combination of the two complementary imaging modes provides specific information about the presence of native and partially degraded GFP-labeled phages, allows them to be distinguished from the auto-fluorescence background and provides a super-resolved image reconstruction of the phage particle localization pattern. SEM imaging of the phage alone and during phagocytosis by macrophages (**F–I**). **(A)** Lambda scan-generated spectra of the enhanced-GFP native form (green curve), the partially degraded form of GFP (red-shifted GFP spectrum, here the red curve), and the auto-fluorescence (magenta curve). **(B)** Typical morphology of the cell loaded with GFP-labeled phages, spectrally unmixed image showing the native-label particles (green), particles with partially degraded GFP label (red) and auto-fluorescence (magenta). Scale bar = 6 μm **(C)** Super-resolution imaging of a representative macrophage with the phage particles. Several clusters of particles visible (GFP-labeled phage). Scale bar = 6 μm. **(D)** Inset showing representative cluster of phages within a macrophage. Scale bar = 1 μm. **(E)** Dimensions of a representative cluster (yellow set) and the estimated measurements of a single phage (red set). The values of particle size approximate the expected dimensions of a phage virus labeled by multiple GFP molecules. Scale bar = 0.6 μm. **(F)** Set of representative macrophages during the uptake of phage particles, SEM scanned at low beam accelerating voltages with SE2 detection at 3 kV acceleration voltage. Scale-bar = 5 μm. **(G)** A representative macrophage during the uptake of phage particles, in lens SE detection at 3 kV acceleration voltage. Scale bar = 1 μm. **(H)** Phage particles on silicon, without macrophages, in-lens SE detection (3 kV). Scale bar = 100 nm. **(I)** Inset showing the uptake of phages into a macrophage, a magnified view of the inset region (red box) in (**G**) in lens SE detection (3 kV). Scale bar = 100 nm.

Phage concentration in the spleen, the major organ responsible for phage clearance^22^,^23^, revealed key differences between SIR mice and normal control mice (Fig. 1A). In SIR mice, the phage concentration was significantly decreased (2.56-log lower, p < 0.05) in spleen. Intensive clearance of phage was linked to a small but significant decrease (1.14-log, p < 0.05) in the number of phage circulating in the blood of the SIR mice shortly (1 hour) after the phage injection. Other tested organs (lymph nodes, kidneys, muscles, liver) did not reveal significant differences (data not shown), indicating organ-specific clearance activity of SIR macrophages (Fig. 1A). Indeed, phagocytes (splenocytes) from SIR mice tested *ex vivo* also inactivated the phage more effectively than those isolated from controls (Fig. 1B). Furthermore, we visualized phage degradation by phagocytosis executed by splenocytes, with super-resolution structural-illumination microscope (Fig. 2) and a green fluorescent protein (GFP)-labeled model phage^24^. The phages were detected within macrophages, typically displayed in groups of GFP-containing particles organized in clusters. The super-resolution imaging was subjected to complementary analysis by spectral unmixing confocal microscopy in lambda model, which was able to identify the pixels of native GFP. This technique showed the ingested phages and co-identified the partially degraded GFP where the pixels displayed red-shifted spectra of GFP (Fig. 2A–E). Thus, we identified phages within macrophage subcellular compartments that were suggestive of the degradation pathway being targeted by bacteriophages in a macrophage.

### Specific immune response to phage

Phage may induce specific antibodies that represent the adaptive arm of immunity^8^,^9^,^10^. To assess the dynamics of this induction, we tested phage-specific IgM and IgG production in mice challenged with the therapeutic *Pseudomonas* phage F8 (Fig. 1C). The primary response (IgM) peaked around 5–10 days after application of the phage, and then declined to a lower level. The secondary response (IgG) followed, and reached the maximum level after the second dose of phage. During the following plateau phase, a high level of anti-F8 IgG antibodies was maintained (Fig. 1C). This profile of humoral responses to the phage fits the typical patterns observed for many other antigens.

Phage circulation in pre-immunized mice was compared to that in naïve mice (Fig. 1D). In pre-immunized mice, phage concentrations in the blood of high IgM animals decreased shortly after phage administration to values 2.52-log lower than in control animals (p < 0.05). The strong secondary response (IgG) had a devastating effect on the phages; no active phages were detected in the blood of animals that had developed a secondary response to the phage, whereas phage concentrations in naïve mice ranged from 8.02-log to 6.36-log. Furthermore, in other tissues notably spleen, liver, kidney, muscle, and lymph node pre-immunization resulted in decreased phage concentrations. The inhibitory effect of IgG appeared to be stronger than that of IgM (Fig. 1D). The key role of antibodies in inactivation of phages in pre-immunized mice was defined *in vitro.* IgM-rich serum reduced the phage activity (1.38-log) and IgG-rich serum inactivated the phage completely (Fig. 1E). Serum complement was identified as a factor contributing to phage neutralization: it weakened the phage viability in the presence of specific antibodies (Fig. 1E). Only highly purified phages (LPS activity less than 0.1 unit per ml) were used. Thus, when the phage degradation pathway was activated, we observed a cooperation of adaptive (antibodies) and innate (complement) elements of the immune system that proved decisive to eliminate the phage activity *in vivo*. The combined antibody/complement effect mimics the mechanism of degradation of eukaryotic viruses^25^,^26^. Here, we demonstrate that phage evokes a similar profile of immune response as eukaryotic viruses do, in spite of the fact that phages do not naturally target mammals.

### Mathematical modeling of reciprocal dependencies

**Figure 3:**
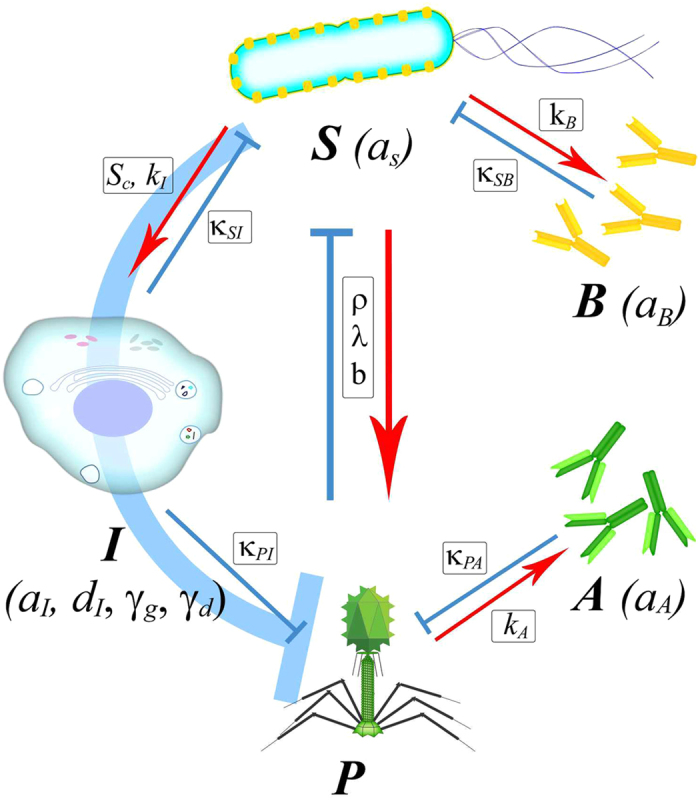
Dynamics involving bacteria, phage, and mammalian immunity *in vivo*. All symbols used in this schema have been applied as variables or parameters in equations of a mathematical model developed for studies of these interactions (see: Materials and Methods) P – bacteriophages, S – bacteria, I – innate immunity, A – adaptive immunity to phages, B – adaptive immunity to bacteria. Red arrows represent a stimulatory while blue arrows represent an inhibitory effect.

Experimental data described above together with previous findings in the field^9^,^27^,^28^ were integrated into a general schema of tripartite interactions between bacteriophages, mammalian immunity and bacteria (Fig. 3) The schema summarizes main reciprocal dependencies, specifically limiting or inducing effects. The key assumptions for this schema were as follows (Fig. 3): first, adaptive immunity specific to phages and adaptive immunity specific to bacteria have no important cross-talk; second, phages are not able to boost innate immunity^27^,^28^; third, boosted innate immunity acts against bacteria, but at the same time it also acts against the phage.

This schema was accommodated into a mathematical model. The model was based on the fundamentals proposed by Levin and Bull^17^,^18^ and Payne and Jansen^19^,^20^, namely on mathematical methods for calculation of phage pharmacokinetics and for theoretical prediction of success or fail of phage therapy. We developed those models with a set of immunology-representing variables as follows: innate immunity (I), adaptive immunity specific to phages (A), and adaptive immunity specific to bacteria (B) (presented in details as equations 1, 2, 3, 4, 5, 6 in the Materials and Methods section).

**Figure 4:**
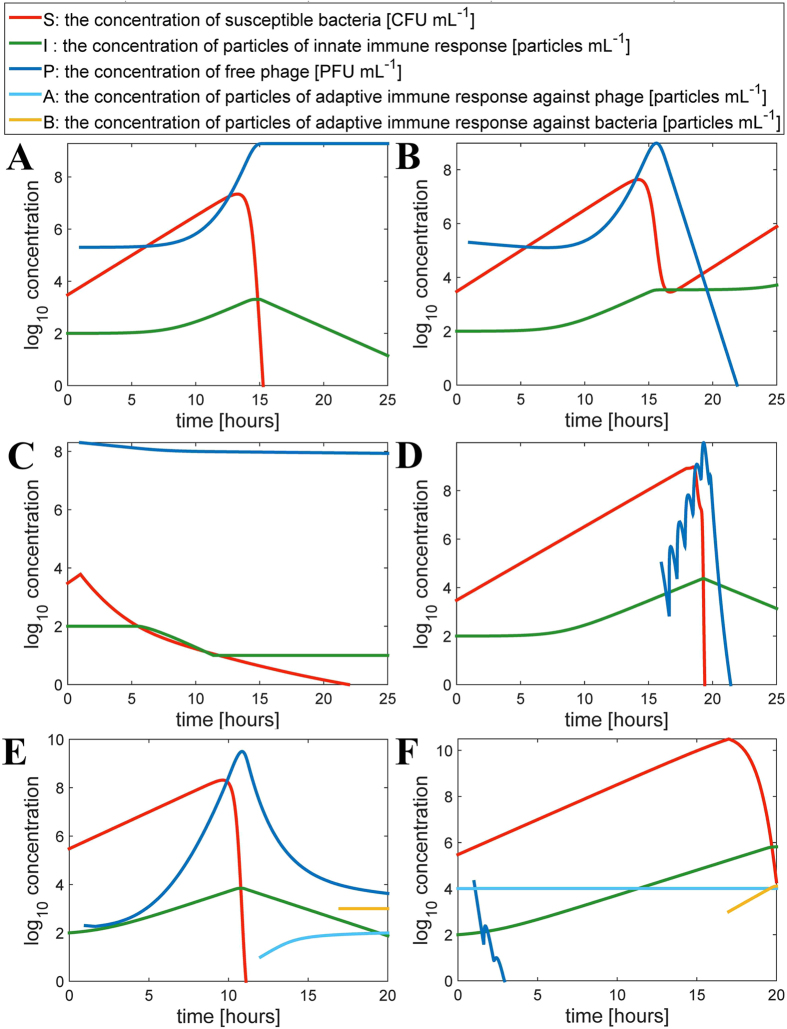
Effect of innate and adaptive immunity on the success or failure of phage antibacterial therapy, numerical simulations. Innate immunity response testing: (**A**) no relation between innate immunity and phage viability accounted, (**B**) phage susceptibility to innate immunity response accommodated, (**C**) phage susceptibility to innate immunity response accommodated and counteracted by increased phage dose, (**D**) phage susceptibility to innate immunity response accommodated and counteracted by a late dose effect. In simulations (**A–D**) values of parameters describing bacterial and phage populations are set to 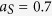, 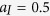, 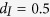, 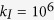, 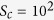, 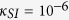, 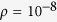, 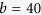, 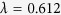 (for units see Table 1). Initial size of the bacterial population is 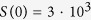. In simulation A 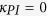 and in other simulations 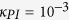. Phage inoculation time is 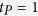 in simulations (**A–C**), and in simulation D 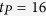. Initial phage inoculation size is 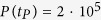 except for simulation (**C**), where 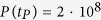. The effect of specific immunization to the phage: (**E**) no specific pre-immunization to the phage exists, (**F**) high level of pre-existing anti-phage antibodies. Simulation in panels E and F is described by the following values of parameters: 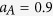, 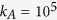, 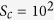, 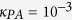, 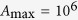, 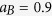, 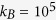, 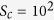, 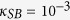, 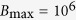. Initial size of the bacterial population is 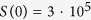. Note that in simulation B phage inoculation size 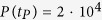 is greater than in simulation A, where 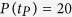. Values of remaining parameters are: 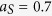, 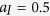, 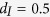, 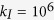, 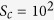, 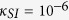, 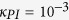, 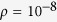, 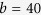

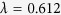
.

This expanded mathematical model when used in numerical simulations demonstrated the key role of the systemic inflammatory response for the outcomes of the therapeutic approach. We simulated therapeutic use of a phage “insensitive” (Fig. 4A) or “sensitive” (Fig. 4B) to the innate immunity response boosted by infecting bacteria; respectively, “success” and “failure” of the phage was observed (Fig. 4A,B). The simulations also allowed for numerical testing and the proposition how to overcome the phage-inhibitory effect of innate immunity. This process can be done by varying the phage dose or by customizing an application schedule (Fig. 4C,D).

In agreement with our laboratory observations (Fig. 1D), numerical simulations also revealed pre-existing immunization to a phage as a major obstacle in systemic antibacterial activity of the phage (Fig. 4E,F). True-life immunization may result from previous therapeutic treatment but also from natural contact with phages that populate the intestine as normal commensal microbiota of humans^29^. Natural immunization can be caused by a similar phage, not necessary by an identical one. Similarities of phages at molecular level relate to similarities in their antigenic determinants and allow for immune cross-reactions of antibodies. Effective immunization may depend on various factors such as individual immunogenic characteristics of a phage, phage dose, time of exposure, route of administration, and others, and according to a recent report it does not necessarily occur as a result of phage therapy^30^. These facts might be important, due to the strong phage-inhibitory effect of high IgG levels in this experimental approach.

## Discussion

Innate immunity is rarely discussed in terms of phage therapy^8^. We showed that in an animal model of systemic inflammatory response, a decrease in availability of active phages in circulation and in selected tissues occurred (Fig. 1A). The mathematical model with the innate immunity-representing variables (equation 2 and 4) demonstrated that the expected outcomes of phage therapy could be abrogated by innate immunity as boosted by bacteria (Fig. 4A,B). This undesirable effect could, however, be counteracted by adjusting the phage dose or altering the timing (Fig. 4C,D), as long as the impact of innate immunity is considered. Bacteria-boosted innate immunity may further explain earlier instances of phage ineffectiveness. The findings also call for the use of other models for phage efficacy testing *in vivo*. Examples could be wound models, serum cytokine evaluations^31^, or molecular imaging of bacterial load in infected organs^32^.

Anti-phage antibody production and the inhibitory effect of humoral (adaptive) immunity on bacteriophages in the mammalian system are widely anticipated, since most examples of phage-immunity reactions apply to the humoral response^9^,^10^,^33^. However, we demonstrated the inhibitory effect of specific antibodies on availability of active phage in the circulation and in numerous selected tissues. Both the primary (IgM) and the secondary (IgG) responses inhibited the phage, but the intensity of this inhibition differed. In the case of high IgG levels, the effect on phage was devastating. No viable phages were detected in murine blood and most tissues while in control mice phage the titers ranged at 10^7^–10^9^ pfu/ml (Fig. 1D). Mathematical modeling showed effective phage therapy in a non-immunized host (Fig. 4E) and ineffective phage therapy in a host with an already existing, significant level of phage-specific antibodies (Fig. 4F). Such antibodies are more likely to be induced by long-term phage treatment. However, they may also result from natural contact with bacteriophages. High levels of anti-phage antibodies obviously increase the risk of failure in a therapeutic approach. Notably, effective specific immunization probably depends on typical factors such as individual immunogenic characteristics of a phage, phage dose, time of exposure, route of administration, accompanying compounds and others and according to a recent report it does not necessarily occur as a result of phage therapy^30^.

Elements of adaptive and innate immunity that inhibit bacteriophages are antibodies (Fig. 1E) and phagocytes (Figs 1B and 2). Interestingly, there was also a link between specific and non-specific action of mammalian immunity on a phage. This interaction was highlighted by the antibody-dependent pathway of serum complement that reduced phage viability (Fig. 1E), similar to well-described inactivation of eukaryotic viruses. Phages lack a lipid outer layer, which is a typical target for the complement, a common feature of eukaryotic viruses. Thus, possible similarities cannot be extrapolated from any eukaryotic viruses, but rather from *Adenoviruses* or adenoviral vectors^26^. The mechanism of complement system interactions with *Adenoviruses* and adenoviral vectors has not been fully determined. The most probable, complement activation *in vivo* involves both classical and nonclassical pathways, as well as the reticuloendothelial system, including removal by hepatic Kupffer cells. Natural immunoglobulin M antibodies opsonize *Adenoviruses*. Complement and opsonins play a contributory role in the clearance of *Adenoviruses* by Kupffer cells. *In vitro* studies are not fully predictive in case of these interactions; however, shielding *Adenoviruses* with polyethylene glycol was effective at reducing complement activation both *in vitro* and *in vivo*^34^,^35^. Good recognition of the mechanism of phage interactions with complement requires further studies.

In summary, we have demonstrated an indirect pathway of phage inhibition by bacteria. This counterintuitive pathway proceeds through innate immunity (Fig. 3, S → I ┤ P), which was stimulated by bacteria, ostensibly for host protection. However, the consequences were phage inhibition. We propose that bacteria can *highjack* the innate immunity of hosts to inhibit phage. This state-of-affairs may determine the results of experimental phage applications in animal models, especially those based on acute septicemia and overt animal morbidity. Furthermore, the immune status of a patient may have a crucial effect on the outcomes of the therapy. We propose that the complexity of mammalian immunity and the *mammalian host-versus-phage* (MHvP) immune response should be taken into account when considering the medical use of phage to combat bacteria.

## Materials and Methods

### Phages, phage cultures, and purification

F8 phage and its *Pseudomonas aeruginosa* host (*P. aeruginosa* F-8) from the IIET Microorganisms Collection were used (Institute of Immunology and Experimental Therapy, Polish Academy of Science Wroclaw, Poland). LB phage cultures were purified by filtration through polysulfone membranes and by fast protein liquid chromatography: gel filtration on Sepharose 4B (Sigma-Aldrich, Poland). The final preparation was dialyzed using 1000 kDa-pore membranes against PBS to remove the bacterial residuals and lipopolysaccharide (LPS), and filtered through 0.22 μm PVDF filters (Millipore, Europe). Fluorescently labeled model bacteriophages were prepared as previously described^24^. Phage titers were detected by routine test dilutions (RTD) on bacterial cultures distributed on the surface of dried agar plates and by applying serial dilutions (50 μl) to phage preparations (in duplicate). The plates were incubated overnight at 37 °C.

### LPS content determination

Endotoxin level of the purified phages was assessed using EndoLISA (ELISA-based Endotoxin Detection Assay, Hyglos, Germany), according to the manufacturer’s instructions. Diluted samples or standard dilution with Binding Buffer were incubated overnight at room temperature with shaking. Subsequently, the plate was washed and Assay Reagent was added. The fluorescent signal was detected immediately in a fluorescence reader (Synergy H4 H4MLFPTAD BioTek Instruments USA). Phages were used as bottom antigens in ELISA or injected into mice only if the LPS content in their preparation was less than 1 unit per ml or per mouse.

### Lipopolysaccharide extraction

*E. coli* was grown for 48 h at 37 °C in LB medium by shaking, then denatured with 0.5% phenol and centrifuged at 39 000 rpm (New Brunswick Scientific, USA). The bacterial pellet was washed three times, lyophilized, treated with 90% phenol/water (1:1), and heated to 65 °C. LPS was extracted according to Westphal and Jann^36^. The extract was cooled to 4 °C, centrifuged at 3000xg for 30 min, and the water phase was collected. Distilled water was added to the remaining phenol phase and the extraction process was repeated. Water phases were combined, dialyzed against water and lyophilized. The final sample was sonicated and diluted to obtain the desired concentrations.

### Animal model

All animal experiments were performed according to EU Directive 2010/63/EU for animal experimentations and were approved by the 1st Local Committee for Experiments with the Use of Laboratory Animals, Wroclaw, Poland (no. 64/2009). The male C57Bl6/J (6–10 weeks) mice were purchased from the Center of Experimental Medicine, Medical University of Bialystok, Poland, and bred under specific pathogen free (SPF) conditions in the Animal Breeding Center of the Institute of Immunology and Experimental Therapy (IIET).

### Immunization of mice

Mice (N = 8) were inoculated intraperitoneally with 0.2 ml of phage (1 × 10^10^ pfu/mouse) on day 0, 20, and 50. Control mice were injected with 0.2 ml of PBS accordingly. Murine blood was collected into heparinized tubes from the tail vein (day 1, 5, 10,15, 20, 25, 30, 35, 40, 45, 52, 55) under anesthesia. Serum was separated from the blood by double centrifugation at 2250 × g and used for the ELISA assay. All experiments were repeated three times. Representative experiments, one for each type, are presented with accompanying statistics.

### F8 phage circulation in mice with systemic inflammatory response

The model of the systemic inflammatory response was developed (see: Supplementary material). Mice were injected intraperitoneally (i.p.) with LPS twice: 2 mg/kg and after 18 hours 1 mg/kg. Control mice were injected with 2.0 ml of PBS. Four hours later all mice were treated i.p. with F8 phage (3 × 10^9^ pfu per mice). After 1, 6, 17 and 24 hours murine blood was collected from the orbital plexus vein into heparinized tubes, under anesthesia. Then animals were sacrificed by cervical dislocation and the following organs were excised: liver, spleen, muscle, kidney, lymph nodes. Organs were homogenized and weighed, and the homogenates were serially diluted with PBS (1 g equaling 1 ml). The phage titer in each tissue/organ was determined by the RTD method.

### F8 phage circulation in pre-immunized mice

Mice were immunized with F8 phage (1 × 10^10^ pfu/mouse) to obtain a high level of IgM and IgG antibodies, according to the immunization schema established in the course of the studies (Fig. 1C) 5 days after the challenge for high IgM level and 20 days after the challenge for high IgG level. High antibody titers were confirmed by ELISA test. Mice were additionally tested for remaining viable phages, and no viable phages were detected. Pre-immunized mice were injected i.p. with F8 phage 3 × 10^9^ pfu/mouse and bled 1, 3,5 hours later from the tail. Seven hours after the phage injection mice were bled from the orbital plexus vein under general anesthesia and sacrificed. Liver, spleen, muscles, kidneys and lymph nodes were excised, homogenized, weighed and serially diluted with PBS (1 g equaling 1 ml). Phage titer was checked by the RTD method.

### Specific antibody level measurement by ELISA

MaxiSorp flat-bottom 96-well plates (Nunc, Thermo Scientific, Europe) were covered overnight with F8 phage (5 × 10^9^ pfu/ml). Subsequently, wells were washed with PBS and blocked with 1% albumin. Diluted serum (1/100 in PBS) was added (100 μl of diluted serum per well). The plate was incubated at 37 °C for 2 hours and washed with 0.05% Tween 20 in PBS (Serva, Europe) 5 times. Diluted detection secondary antibody was applied (100 μl per well): peroxidase-conjugated AffiniPure goat anti-mouse IgM (Jackson ImmunoResearch Laboratories) or peroxidase-conjugated AffiniPure goat anti-mouse IgG (Jackson ImmunoResearch Laboratories). The plate was incubated for 1 h at room temperature in the dark. DuoSet of substrate reagents for peroxidase was used according to the manufacturer’s instructions (DY999, R&D Systems, Europe) and incubated for 20 minutes. Twenty-five μl of 2 N H_2_SO_4_ was added, and absorbance was measured at 450 nm (main reading) and 570 nm (background).

### Phage blocking by specific sera

Serum obtained from pre-immunized mice on the 5^th^ day (IgM-rich serum) and 20^th^ day (IgG-rich) after injecting F8 phage (1 × 10^10^ pfu/mouse) was used. As a control, serum from PBS-injected mice was used. Each serum sample was applied as an inactivated or non-inactivated serum (i.e. after or without inactivation of serum complement). Serum was heat-inactivated by incubation at 56 °C for 1 h. For blocking of phage activity, F8 phage (10^9^ pfu/ml) was mixed with serum samples (1:1) and incubated at 37 °C for 1.5 h. Phage titers in the samples were determined by the RTD method. Seven samples per group (N = 7) were tested, and the assay was repeated three times. An example experiment with individual statistics is presented.

### Bacteriophage phagocytosis by splenocytes

Fractionating isolation of splenocytes was conducted according to Kruisbeek^37^ and Boyum^38^ with modifications. Briefly, two groups of mice (N = 7) were used: mice with an SIR and normal mice. An SIR was induced by LPS as established (see: Supplementary material). The mice were injected intraperitoneally with LPS twice (2 mg/kg and after 18 hours 1 mg/kg). Control mice were injected with 2.0 ml of PBS. Four hours later all mice were sacrificed, and spleens were excised and homogenized. The cells were isolated in a density gradient with Histopaque 1083 (Sigma-Aldrich) by centrifugation (700 × g, 30 min, 20 °C). Then the mononuclear cell layer of Histopaque 1083 was collected, and the cells were washed three times (5 min, 870 × g, 4 °C) in PBS. Morphology, quantity and general quality of the isolate were verified by optical microscopy in the histological Bürker chamber. The cells were then diluted in Hanks solution supplemented with 5% fetal calf serum (FCS) to achieve the required cell density for the further experiments.

Phagocytosis was tested in 24-well plates. Three groups (N = 5) of cells were tested: SIR cells, normal cells, and normal cells induced by PMA (phorbolmyristate acetate, 50 ng/ml). Cells were used in a final density of 10^6^ cells/ml. F8 phage was added to a final count of 10^5^pfu/ml (volume: 1 ml). Cultures were incubated for 2 h at 37 °C, in 5% CO_2_. Samples were centrifuged (200 × g, 4 °C, 5 min) and viable phage titer was determined by RTD in the culture supernatant. Each experiment was repeated twice. Anexemplary experiment is presented in the figure.

### Imaging of bacteriophage uptake by macrophages with spectral unmixing confocal microscopy and super-resolution structural illumination microscopy

Prior to high-resolution imaging of viral particles, the enhanced green fluorescent protein (EGFP) signal was identified by spectral unmixing from the auto-fluorescence background using the confocal microscopy system equipped with an array of simultaneously recording 32-channel spectral detectors. Image frames were collected using a confocal microscope (LSM 780, Carl Zeiss) equipped with an LSM780 GaAsP detector, using a Plan-Apochromat 100 × oil-immersion objective with numerical aperture 1.46, Carl Zeiss, Jena, Germany, analysis of data being performed with ZEN 2012 software. The spectra recorded from the sample were compared to known EGFP spectra, both native and partially catabolized (red-shifted), to detect intact and partially degraded EGFP of bacteriophages. The auto fluorescence was spectrally analyzed, and the three major pools of fluorescence, the native EGFP, partially degraded EGFP and the auto fluorescence spectra were unmixed. After confirmation that macrophages express EGFP of expected spectral characteristics, high-resolution imaging was performed using the structural-illumination method of super-resolution microscopy.

Super-resolution microscopy by the structured illumination approach was used for imaging of phages applied on macrophages. The uptake of bacteriophages by macrophages was tested after stimulation with bacterial LPS and compared to control conditions under which PBS was used instead of LPS in mice (the donors of macrophages). The EGFP-containing construct was used for detection of viral particles. T4 phages were labeled by incorporation of EGFP-Hoc fusions into T4ΔHoc particles, as described above.

Super-resolution structured illumination (SR-SIM) was performed. Thin (0.1 μm) Z-layer stacks of high-resolution image frames were collected in 5 rotations by using an alpha Plan-Apochromat 100×/1.46 oil DIC M27 ELYRA objective, using an ELYRA PS.1 tandem microscopy system (Carl Zeiss, Jena, Germany) equipped with a 488 nm laser (100 mW), 561 nm laser (100 mW) and Andor EM-CCD camera (iXon). Images were reconstructed using ZEN software (black edition, 2011) based on a structured illumination algorithm. Analysis was performed on the volume-rendered super-resolution images in ZEN.

Scanning Electron Microscopy (SEM) of bacteriophage particles alone and phage uptake process by macrophages were processed without any coating. The viral particles suspension, alone or mixed together with macrophages, have been applied to silicon chip and allowed to adhere for 30 minutes at 37 degrees Celsius in 5% CO_2_/air incubator in PBS supplemented with calcium and magnesium chloride. The samples have been fixed with 2.5% glutaraldehyde in 0.1 M cacodylate buffer for 30 minutes at 4 degrees Celsius, then washed, dehydrated in series of methanol (25%–50%–75%–100%–100%) in 1 hour steps at 4 degrees Celsius. Samples underwent critical point drying with methanol exchanged for liquid CO_2_in an automatized manner, (CPD300 AUTO, Leica Microsystems, Austria) and imaged with cross-beam scanning electron microscope equipped with Schottky field-emission cathode (Auriga 60, Carl Zeiss, Oberkochen, Germany) at 3 kV accelerating voltage. Images represent the Everhart-Thornley or in-lens SE detection from the sample surfaces, with no coating or contrasting applied

### Mathematical model

This model was developed from the models of Levin and Bull^17^,^18^ and Payne and Jansen^19^,^20^ (for extensive description of methodology see: Supplementary material). Dynamics of bacteria, phage and immune system interactions are described by the system of delay differential equations (DDE):


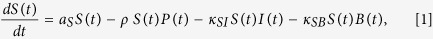



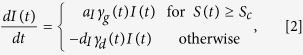


where


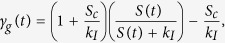


and


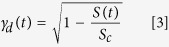



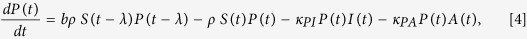



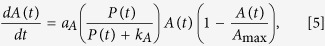



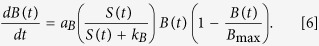


State variables and parameters of the model are as follows:[Table t1]

## Additional Information

**How to cite this article**: Hodyra-Stefaniak, K. *et al.* Mammalian Host-Versus-Phage immune response determines phage fate *in vivo*. *Sci. Rep.* **5**, 14802; doi: 10.1038/srep14802 (2015).

## Supplementary Material

Supplementary Information

## Figures and Tables

**Table 1 t1:** 

**State variable**		
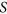	concentration of susceptible bacteria	[cfu mL^−1^]
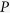	concentration of free phage	[pfu mL^−1^]
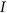	concentration of particles of innate immune response	[particles mL^−1^]
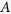	concentration of particles of adaptive immune response against phage	[particles mL^−1^]
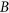	concentration of particles of adaptive immune response against bacteria	[particles mL^−1^]
**Parameters**		
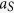	growth rate of susceptible bacteria	[h^−1^]
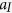	growth rate of innate immune response	[h^−1^]
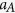	growth rate of adaptive immune response against phage	[h^−1^]
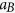	growth rate of adaptive immune response against bacteria	[h^−1^]
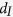	decay rate of innate immune response	[h^−1^]
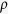	adsorption rate of phages by susceptible bacteria	[mL cfu^−1^ h^−1^]
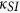	killing rate of bacteria versus innate immune response	[h^−1^]
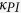	killing rate of phage versus innate immune response	[h^−1^]
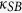	killing rate of bacteria versus adaptive immune response	[h^−1^]
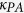	killing rate of phage versus adaptive immune response	[h^−1^]
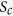	bacterial concentration above which innate immune response increases	[cfu mL^−1^]
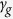	growth rate reduction of innate immune response	[–]
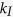	bacterial concentration at which innate immune response actual growth rate is half of its maximum value	[cfu mL^−1^]
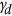	decay rate reduction of innate immune response	[–]
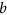	phage burst size	[pfu]
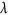	latent period (average time between phage adsorption and burst)	[h]
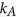	phage concentration at which adaptive immune response actual growth rate is half of its maximum value	[pfu mL^−1^]
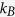	bacterial concentration at which adaptive immune response actual growth rate is half of its maximum value	[cfu mL^−1^]
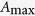	maximum magnitude of 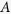	[particles mL^−1^]
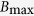	maximum magnitude of 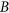	[particles mL^−1^]
