# Supplementary material for: Mammalian Host-Versus-Phage immune response determines phage fate in vivo
Source: Sci Rep. 2015 Oct 6;5:14802. doi: 10.1038/srep14802 (PMC4594097; doi:10.1038/srep14802)
Supplement: Supplementary Information [file srep14802-s1.pdf]

Supplementary information:

Mammalian Host-Versus-Phage immune response determines phage fate in vivo

Katarzyna Hodyra-Stefaniak[1], Paulina Miernikiewicz[1], Jarosław Drapała[2], Marek Drab[3], Ewa Jończyk-Matysiak[1], Dorota Lecion[1], Zuzanna Kaźmierczak[1], Weronika Beta[1], Joanna Majewska[1], Marek Harhala[1], Barbara Bubak[1], Anna Kłopot[1], Andrzej Górski[1], Krystyna Dąbrowska[1*]

Murine model of systemic inflammatory response

Lipopolysaccharide (LPS) is a potent stimulator of inflammatory marker production[39], able to induce an SIR. It is also a typical 'alert' signal for an immunological reaction in case of infection with Gram-negative bacteria. Typical markers of inflammation that are induced by LPS are cytokines, e.g. TNF-α, IL-1 and IL-6. We used LPS to induce a systemic inflammatory reaction in C57Bl6/J mice (two doses of LPS: the first dose of 2 mg/kg and 18 hours later the second dose of 1 mg/kg). Control mice were treated with PBS, accordingly. Four hours after the second dose of LPS, TNF-α, IL-1 and IL-6 were quantitated in murine blood. A significant increase of all investigated cytokines in the blood was observed in LPS-treated mice in comparison to the control. The increase of TNF-α was 4.3-fold, IL-1 was increased 6.07-fold and IL-6 was increased 3.12-fold (Supplementary Fig. 1). Markedly increased inflammatory markers in the blood confirm a systemic reaction to LPS and stimulation of innate immunity. This model was applied for testing phage circulation in conditions of an SIR.

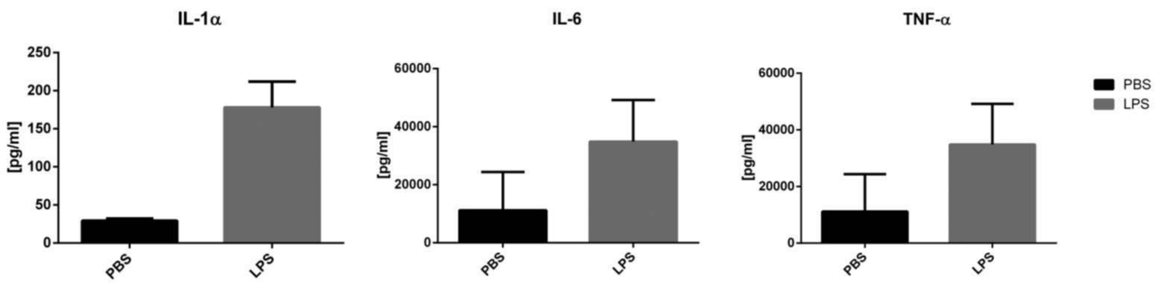

Supplementary Figure 1. Inflammatory markers in murine model of systemic inflammatory response (SIR).

C57Bl6/J mice were injected intraperitoneally with two doses of LPS: the first dose of 2 mg/kg and 18 hours later the second dose of 1 mg/kg. Control mice were treated with PBS, accordingly. Four hours after the second dose TNF-α, IL-1 and IL-6 was quantified in murine blood by quantitative development ELISA test.

LPS – the level of inflammatory markers in mice treated with LPS; PBS – the level of inflammatory markers in control mice treated with PBS.

Mathematical model

We modified and extended the models of Levin and Bull[17, 18] and Payne and Jansen[19,20] as described in the Material and Methods section. Equation (1) describes the growth of the bacterial population. The number of bacterial cells grows exponentially, unless the growth is reduced by phage, or the innate or adaptive immune system. Consequently, interaction terms in equation (1) all have minus signs. We assume that the decrease of the growth rate is proportional to both the current size of the bacterial population and the current size of antagonist populations.

Equations (2)-(3) constitute our proposed model of the innate system response. When the concentration of bacterial cells exceeds the threshold S_c and resources are unlimited, the

concentration of innate system particles grows exponentially with a constant rate a_I. Functions γ_g, γ_d are modifications of the hyperbolic function proposed by Monod[33]:

$$\gamma(t) = \frac{C(t)}{C(t)+k}, \qquad (7)$$

where C stands for the amount of a resource and k is the value of C for which γ is half of its maximum value. Note that γ takes values from the range $\langle 0,1 \rangle$. We say that formula (7)has one degree of freedom, because we may set up the value of one parameter k in order to fit the formula to laboratory data.

Those functions describe the relation between growth rate reduction of a cell population and resource concentration. From the immune system and phage point of view, the bacteria are a resource. Therefore, actual growth and decay rates of the innate system are always lower than a_I and d_I, respectively. This is expressed by multiplication of a_I by γ_g or d_I by γ_d. The actual growth and decay rates of the innate immune response are equal to $a_I\,\gamma_g(t)$ or $d_I\,\gamma_d(t)$.

We decided to extend formula (7) to formula (3), having one additional degree of freedom S_c. Moreover, formula (3) has discontinuity of the first derivative at point S_c. The general idea behind formula (3) is to allow the innate system response to decay, which is not the case in the Monod[40] formula (7). The comparison between the Monod function and our extension is given in Supplementary Figure 2. It may be considered, on the basis of the theoretical background or laboratory observations, whether function (3) should be smooth at the critical point S_c.

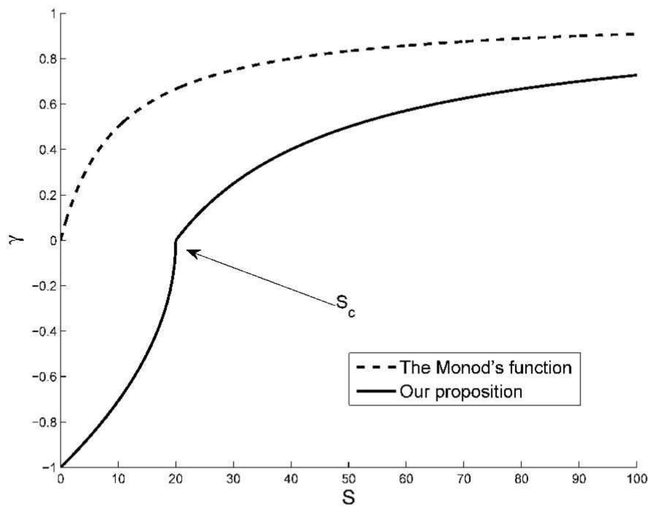

Supplementary Figure 2. Illustration of formula (3).

Dynamics of a free phage population are described by equation (4). The second term also appears in equation (1). This is the amount of bacteria-phage units that have arisen as a consequence of phage adsorption at the current time. Adsorbed phages can no longer infect other cells, hence the minus sign. The first term has a similar form as the second except that it is delayed by λ time units. Its role is to take into account new free phages that were released by bacterial cells infected λ hours ago. It is assumed that bacteria and the phage are excluded from their populations immediately after coupling. The two terms described here come from the work of Levin and Bull[18]. We neglect the so-called thermodynamic effects, which within a time cause degradation of free phage. Instead, we included two additional terms describing phage loss due to innate and adaptive immune responses.

Dynamics of adaptive immune responses against phage and against bacteria are modeled using equations (5) and (6), respectively. These equations have a similar form to the equation proposed by Levin and Bull[18] for the immune system. The last factor that we included in both equations converts the exponential growth (assumed for the innate response) into logistic growth. Note that in the literature, innate and adaptive immune responses are not

clearly distinguished, whereas we treat them as two different systems. There are two important differences between innate and adaptive responses that our model takes into account: onset time (the innate response precedes adaptive response activation) and scope (the innate response suppresses both bacteria and phage, whereas the adaptive response is specific). According to the current state of knowledge[27], we assume that only bacteria stimulate the innate response, while the phage has no effect on innate immunity. However, innate immunity affects both bacteria and phages. In the case of bacteria whose propagation depends on resources delivered by a mammalian host, in most cases bacteria-innate immunity dependency resembles the typical predator-prey pattern, i.e. strong stimulation of innate immunity (systemic inflammatory response) by a large number of bacteria eventually inhibits bacteria; reduction of this pressure (low bacterial number) allows for bacterial growth. Additional action of adaptive immunity can lead to destruction of bacteria. In the case of bacteria that intensively stimulate the systemic inflammatory response but are not very sensitive to the action of innate immunity, the results can be fatal to the mammalian host (Supplementary Fig. 3).

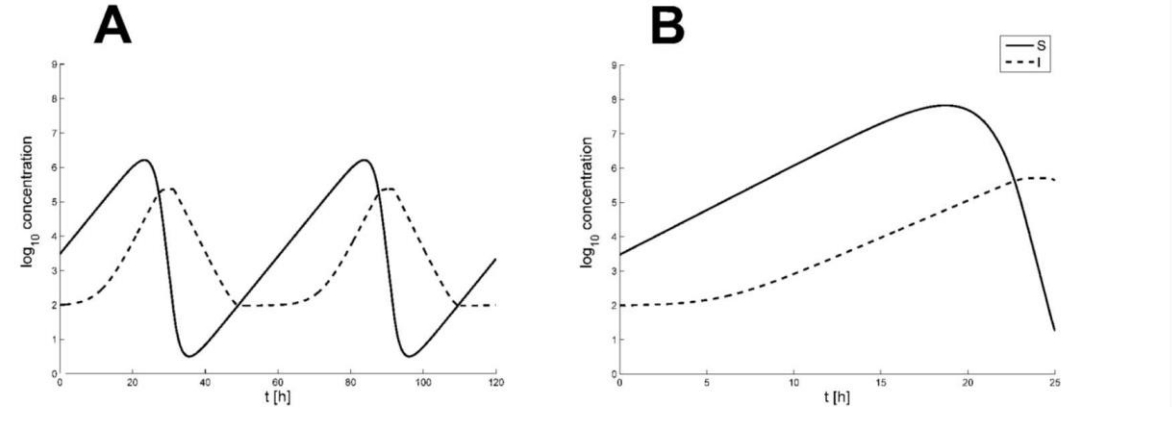

Supplementary Figure 3. Interaction of bacterial population and innate immune response. In both simulations initial size of bacterial population is $S(0) = 3 \cdot 10^3$ and values of parameters describing innate immune response are $a_I = 0.5$, $d_I = 0.5$, $k_I = 10^5$, $S_c = 10^2$,

$\kappa_{SI} = 10^{-5}$. The only difference concerns the growth rate, which equals $a_S = 0.3$ in panel A and $a_S = 0.5$ in panel B.

Computer simulations were performed in MATLAB r2014a with the use of ode45 and dde23 solvers[41]. In all simulations we assume that the critical level of bacterial concentration is 10^9. When the bacterial concentration is higher, it means that the infection cannot be overcome by the host response (the lethal effect).

Supplementary References:

39. Park, B.S,, Song, D. H., Kim, H. M., Choi, B. S., Lee, H., Lee, J. O. The structural basis of lipopolysaccharide recognition by the TLR4-MD-2 complex. *Nature.***458**, 1191-1195 (2009).

40. Monod, J. The growth of bacterial cultures. *Ann. Rev. Microbiol.* **3**, 371-394 (1949).

41. Shampine, L. F., Gladwell, I., Thompson, S. S*olving ODEs with MATLAB.* (Cambridge University Press, United Kingdom, 2003).
